# 17beta-estradiol counteracts neuropathic pain: a behavioural, immunohistochemical, and proteomic investigation on sex-related differences in mice

**DOI:** 10.1038/srep18980

**Published:** 2016-01-08

**Authors:** Valentina Vacca, Sara Marinelli, Luisa Pieroni, Andrea Urbani, Siro Luvisetto, Flaminia Pavone

**Affiliations:** 1 CNR-National Research Council, Institute of Cell Biology and Neurobiology, 00143 Roma, Italy; 2 IRCCS Fondazione Santa Lucia, 00143 Roma, Italy; 3 Department of Experimental Medicine and Surgery, Division of Biochemistry, University of “Tor Vergata”, 00133 Roma, Italy

## Abstract

Sex differences play a role in pain sensitivity, efficacy of analgesic drugs and prevalence of neuropathic pain, even if the underlying mechanisms are far from being understood. We demonstrate that male and female mice react differently to structural and functional changes induced by sciatic nerve ligature, used as model of neuropathic pain. Male mice show a gradual decrease of allodynia and a complete recovery while, in females, allodynia and gliosis are still present four months after neuropathy induction. Administration of 17β-estradiol is able to significantly attenuate this difference, reducing allodynia and inducing a complete recovery also in female mice. Parallel to pain attenuation, 17β-estradiol treated-mice show a functional improvement of the injured limb, a faster regenerative process of the peripheral nerve and a decreased neuropathy-induced gliosis. These results indicate beneficial effects of 17β-estradiol on neuropathic pain and neuronal regeneration and focuses on the importance of considering gonadal hormones also in clinical studies.

A higher prevalence of pain conditions, acute as well as chronic, together with higher susceptibility to nociceptive stimuli and more frequent use of analgesic medications is reported in women than in men^1^,^2^,^3^. Moreover, women show a heightened inflammatory response compared to men^1^,^4^,^5^, supporting a direct contribution to inflammation played by estrogens in pain^6^.

Estrogens regulate a large spectrum of neuronal functions including pain^7^,^8^,^9^,^10^. The administration of estrogens may induce pro- or anti-nociceptive effects, depending on dose and on animal model of pain considered^11^,^12^. These effects are only in part mediated by a direct action of estrogens on neurons. In addition, estrogens regulate the function of the nervous system by acting on glial cells^13^,^14^, which are involved in a large variety of functions, including the regulation of neuronal metabolism, neuronal activity, plasticity and neural regeneration^15^,^16^. Therefore, the action of estrogens on glia is important to maintain physiological homeostasis, to modulate cellular products and proliferation of SC, and to regulate myelination and remyelination processes.

The physiological action of estrogens is exerted by different mechanisms through which the three estrogens receptors (ERs), ERα, ERβ^17^ and the estrogen G-protein coupled receptor GPR30, mediate genomic and non-genomic actions^18^. It has been demonstrated that ERs are widely and differently distributed throughout central peripheral nervous systems^19^,^20^; peripheral sensory neurons express both ERα and ERβ, with ERα being selectively localized on small-diameter sensory neurons^21^.

In a previous study we demonstrated significant sex-related differences in the development and recovery from neuropathic pain in mice. Male mice subjected to chronic constriction injury (CCI) of the sciatic nerve showed a gradual and progressive decrease of allodynic response and a complete recovery^22^. On the other hand, in female mice, CCI-induced allodynia was still present 121 days after nerve ligation. The regenerative process consequent to the injury was faster in males than in females and was supported by different expression of proteins associated with nerve injury and repair.

Although the beneficial effect of estrogens therapy in human are still under debate, a vast literature shows that 17β-estradiol is neuroprotective, have anti-inflammatory effects on nervous system and strongly influences neuroimmune communication pathways^23^,^24^,^25^. A strong sexual dimorphism exists as part of the immune response, and estrogens are responsible, in part, for many sex differences^6^,^13^.

The goal of this study was to evaluate if the administration of 17β-estradiol was able to reduce neuropathic pain, recover hindlimb functionality and affect regenerative processes and glial cells influence. Sex-related differences in these responses were examined by using behavioral investigations, immunofluorescence staining, and proteomic analysis.

## Results

### Behavioral testing

Behavioral responses were examined to evaluate the effects of 17β-estradiol treatment on mechanical allodynia and functional recovery in neuropathic female and male mice.

#### Mechanical nociceptive threshold

**Figure 1:**
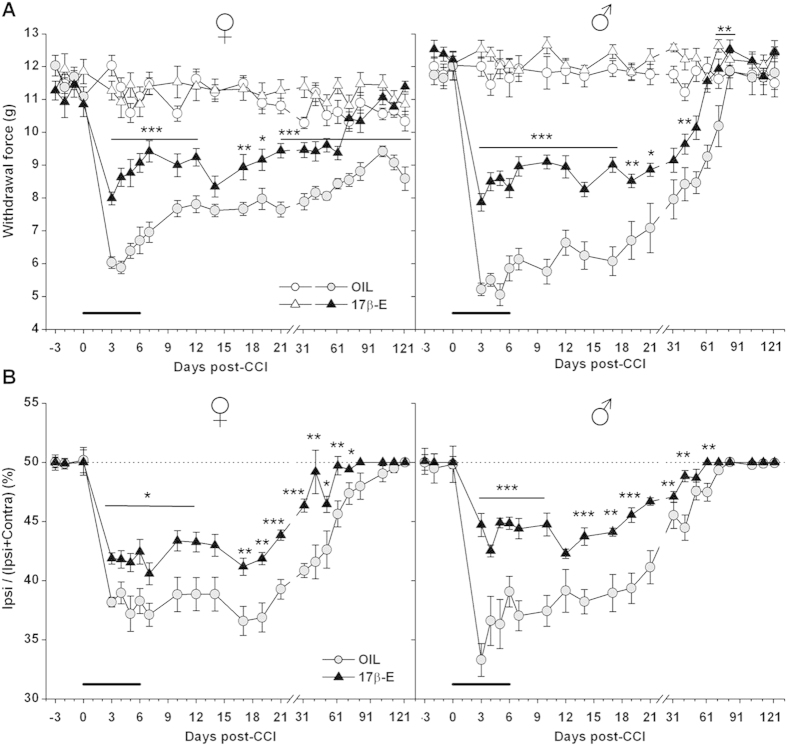
Behavioral responses to neuropathic pain in female and male mice (A) *Mechanical nociceptive threshold.* Sex-related differences in the development of mechanical allodynia induced by Chronic Constriction Injury (CCI): time course of withdrawal thresholds (expressed as applied force in grams) of hindpaws ipsi- and contralateral to the injury in OIL (ipsi 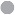, contra ○) and 17β-estradiol (17β-E) (ipsi ▲, contra △), female (left panels) and male (right panels) mice. Two-way ANOVAs for repeated measures showed significant differences for treatment (F_1,18_ = 101.268, P < 0.0001 and F_1,18_ = 106.527, P < 0.0001, for females and males respectively), time (F_19,342_ = 25.273, P < 0.0001 and F_19,342_ = 56.858, P < 0.0001, for females and males respectively) and for treatment x time interaction (F_19,342_ = 2.835, P < 0.0001 and F_19,342_ = 3.764, P < 0.0001, for females and males respectively). Number of mice was 10 for each experimental group. (*)P < 0.05; (* *)P < 0.01; (***)P < 0.001 vs OIL. (**B**) *Weight bearing.* Weight percent distribution time course of the hind limb ipsilateral to the injury in OIL (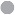) and 17β-estradiol (17β-E) (▲), female (left panels) and male (right panels) mice. Two-way ANOVAs for repeated measures showed significant differences for treatment (F_1,18_ = 17.823, P < 0.0005 and F_1,18_ = 20.498, P < 0.0005 for females and males respectively), time (F_19,342_ = 62.9641, P < 0.0001 and F_19,342_ = 70.249, P < 0.0001 for females and males respectively) and treatment x time interaction (F_19,342_ = 2.932, P < 0.0001 and F_19,342_ = 11.045, P < 0.0001 for females and males respectively). Number of mice was 10 for each experimental group. (*)P < 0.05; (* *)P < 0.01; (***)P < 0.001 vs OIL.

Figure 1A shows the mechanical nociceptive thresholds in OIL- (vehicle) and 17β-estradiol- (17β-E) treated female and male mice after CCI. Sciatic nerve ligation induced allodynia in both sexes but with different time courses in female and male groups. In vehicle-treated CCI females, mechanical nociceptive threshold decreased by about 50% in the ipsilateral compared to contralateral hindpaw. 17β-estradiol induced a significant reduction in allodynia in female mice, showing a higher withdrawal threshold for the injured paw compared to OIL female group during the overall time-course, starting from the third day after CCI. The complete recovery of this group was observed by day 71 (D71) after CCI. In accord with our previous study^22^, in OIL female mice lack of recovery from neuropathy was observed. It is noteworthy the efficacy of 17β-estradiol in inverting this tendency and in making females able to recovery.

Figure 1A shows that also in males the time course of allodynic response is different depending on treatment. Starting by D3, 17β-estradiol male group showed a higher withdrawal threshold for the injured paw in comparison with OIL-treated males till D31, a complete recovery occurring 61 days after injury. Treatment with 17β-estradiol facilitated recovery and supported a faster regenerative process, as demonstrated by the following immunohistochemical results. OIL male groups showed a gradual reduction of CCI-induced allodynia only starting by D31, with the complete recovery occurring 81 days after CCI: twenty days later than 17β-estradiol-treated males.

These data show that the activation of estrogen receptors are involved in neuropathic processes and are able to induce analgesia both in females and males and to facilitate the recovery after injury.

To exclude potential effects of 17β-estradiol on locomotor activity, preliminary experiments were carried out in all animal groups at D7 post CCI: 17β-estradiol did not affect locomotor activity neither in females nor in male mice (See Supplementary Fig. S1).

#### Weight bearing (incapacitance test)

The results obtained by using incapacitance test highlight sex-related differences in response to injury and the facilitating effects of 17β-estradiol on functional recovery.

The body weight distribution on the hindlimbs is unbalanced after CCI (Fig. 1B). In female groups at D3 there was a weight shift from the ipsilateral to the contralateral hindlimb. A more balanced weight distribution was observed in 17β-estradiol females than in OIL female mice. From D19 post-CCI, 17β-estradiol females started to normalize their weight bearing and, at D51 from CCI, an equal weight distribution on the hindlimbs was reached. For OIL group the functional recovery developed more slowly, being a complete balance between the two hind limbs at D101.

On D3 from CCI, there was a weight shift also in male groups (Fig. 1B). This unbalance was lesser in 17β-estradiol group, which showed a faster normalization of weight bearing. The complete balance in 17β-estradiol mice occurred 61 days after CCI, while in OIL males 71 days after.

### Estradiol plasma levels

The measurement of circulating estradiol in the experimental groups revealed a higher level of estradiol in naïve females in comparison with male mice and a decrease of the hormone level after neuropathy induction in females more than in males. This reduction was counteracted by 17β-estradiol treatment that induced in female mice a significant enhancement of estradiol level (see Supplementary Fig. S2).

### Immunohistochemical analysis

We analyzed the expression of some proteins markers associated with peripheral nerve de/regenerative processes and with spinal glial activation in neuropathic animals to verify the effects of 17β-estradiol on these structural processes and for a correlation with its functional activity.

#### Sex differences in the expression of ERα and S100β on sciatic nerve

**Figure 2:**
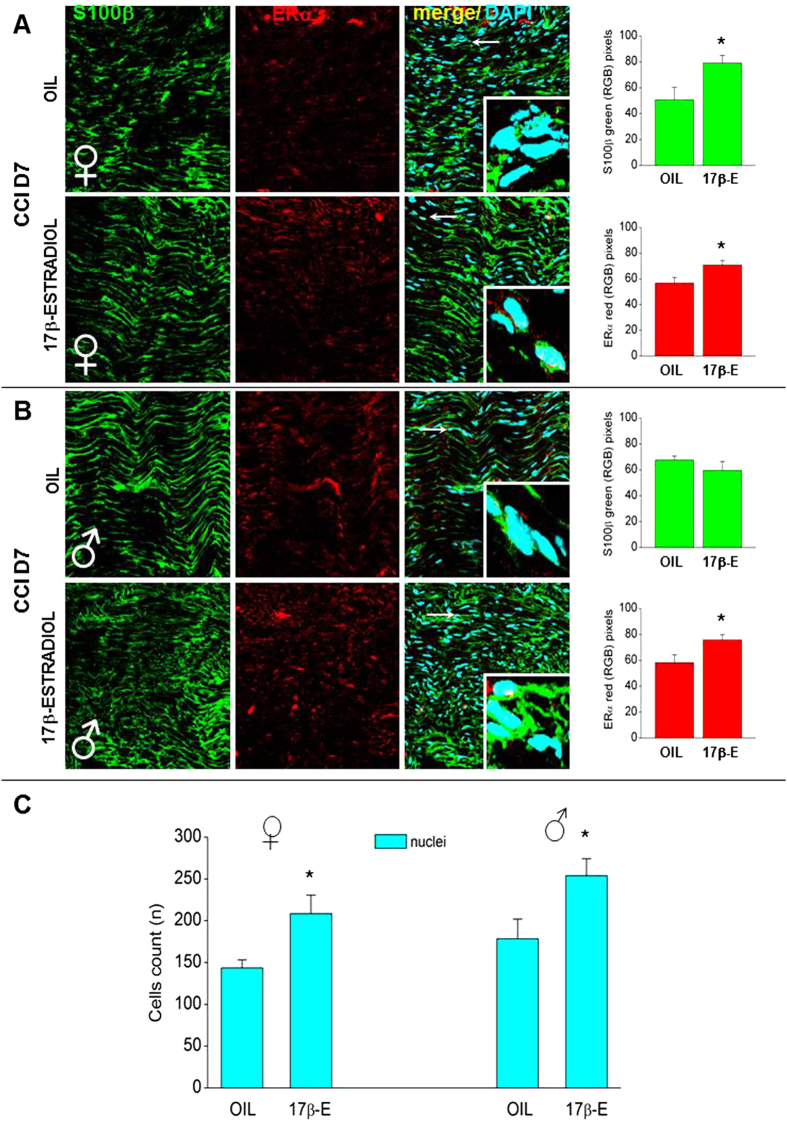
Sex-dependent differences in the expression and quantification of ERα, S100β and nuclei in sciatic nerves at CCI D7. (**A,B**) Representative examples of high magnification (63X) IF images of S100β (green), ERα (red) and colocalization between S100β and ERα (merge, yellow), taken from sciatic nerves of OIL or 17β-estradiol female (**A**) and male (**B**) mice. Scale bar: 50 μm. White arrows indicate areas shown in the inset squares (63x, digital zoom 3). Right panels show quantification of S100β and ERα in OIL and 17β-estradiol (17β-E) mice by using RGB method that converts pixel in brightness values. (*)P < 0.05 vs OIL. (**C**) Histograms show the total number of nuclei (Hoechst positive cells) in OIL and 17β-estradiol female and male mice. (*)P < 0.05 vs OIL.

To analyze the role of estrogens in remyelination after injury, IF analysis was performed with antibodies against S100β, a marker of SC, and ERα, a marker of Estrogen-related Receptor α. IF analysis, made at D7 after CCI (CCI D7) shows a colocalization of ERα staining with S100β-positive cells (Fig. 2).

A first staining was made before injury to verify sex-related differences in the distribution of ERα on sciatic nerve fibers. In female and male mice without injury, nerves were mostly homogenous and ERs were visible along the fibers (S100β-positive), in particular near the node of Ranvier. The quantification of Hoechst positive cells and the analysis with RGB method, confirmed a similar expression and distribution of ERα (females: 32.01 ± 6.08; males: 37.40 ± 2.99) and S100β (females: 68.192 ± 5.98; males: 65.06 ± 9.94) in both sexes.

In proximal area to CCI, there was an increase in S100β and ERα immunoreactivity in 17β-estradiol females in comparison with OIL females (Fig. 2A). The higher magnification confirmed the clear ERα staining associated with SC profile, as revealed by the colocalization of ERα with S100β. Double-immunostaining on cross sections showed a higher ERα expression around nuclei of SC (see the inset square of high magnification IF images, 63x, digital zoom 3). In OIL CCI female mice the delayed functional recovery could be caused by reduced ability of SC to proliferate and guide the outgrowing axons^26^,^27^. These data were confirmed by the quantification of Hoechst positive cells: after CCI, we observed a remarkable increase in 17β-estradiol compared to OIL females (Fig. 2C). The evaluation of ERα and S100β expression with RGB method confirmed differences between the two groups (Fig. 2A, histograms): in 17β-estradiol females a higher expression of ERα and S100β was observed.

In male mice, injury induced an increase of ERα expression in 17β-estradiol group compared to OIL group (Fig. 2B). The higher magnification shows, as already observed in females, the colocalization between ERα and S100β. The brightness evaluation by means of RGB method confirmed the higher expression of ERα in 17β-estradiol males. Moreover, males treated with 17β-estradiol show an increase of Hoechst positive cells compared with OIL males (Fig. 2C). The enhancement of SCs proliferation may induce local axonal sprouting, known to occur during regeneration.

#### Sex differences in the expression of Neu200 and P0 on sciatic nerve

**Figure 3:**
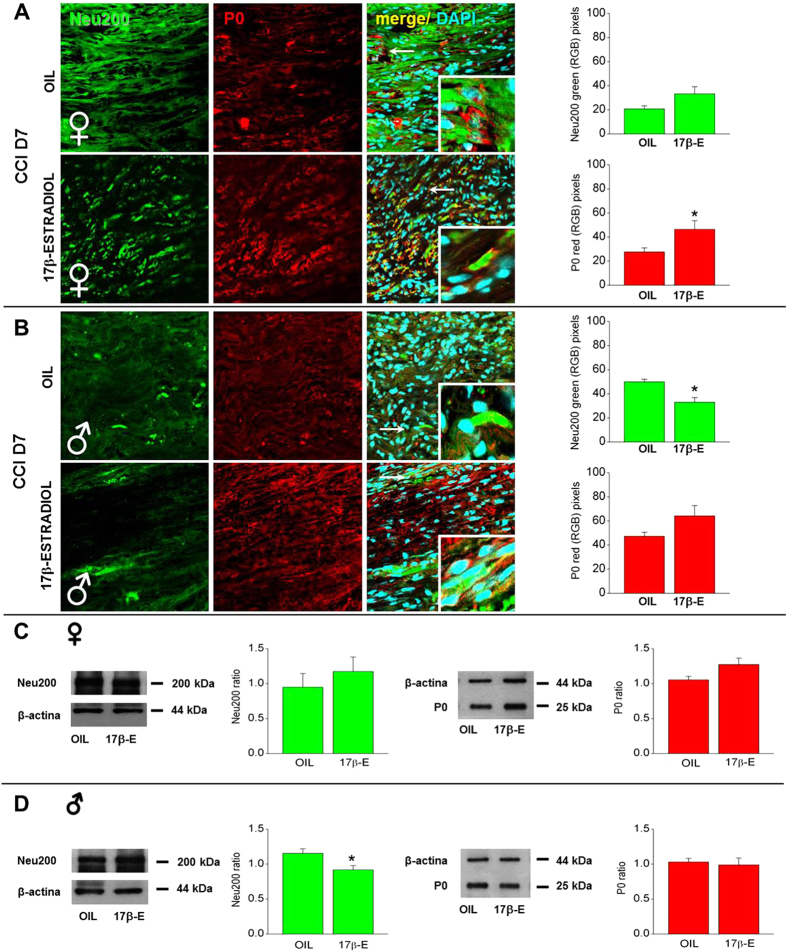
Sex-dependent differences in the expression and quantification of Neu200 and P0 in sciatic nerves at CCI D7. (**A,B**) Representative examples of high magnification (63X) IF images of Neu200 (green), P0 (red) and colocalization between Neu200 and P0 (merge, yellow), taken from sciatic nerves of OIL and 17β-estradiol female (**A**) and male (**B**) mice. Scale bar: 50 μm. White arrows indicate areas shown in the inset squares (63x, digital zoom 3). Right panels show quantification of Neu200 and P0 in OIL and 17β-estradiol (17β-E) mice by using RGB method that converts pixel in brightness values. (*)P < 0.05 vs OIL. (**C,D**) Representative examples of Western blot analysis of Neu200 and P0 protein expression in sciatic nerves from OIL or 17β-estradiol (17β-E) female (**C**) and male (**D**) mice. Histograms show quantification of Neu200 (green) and P0 (red) as obtained using the software ImageJ software (version 1.41, National Institutes of Health, USA).

**Figure 4:**
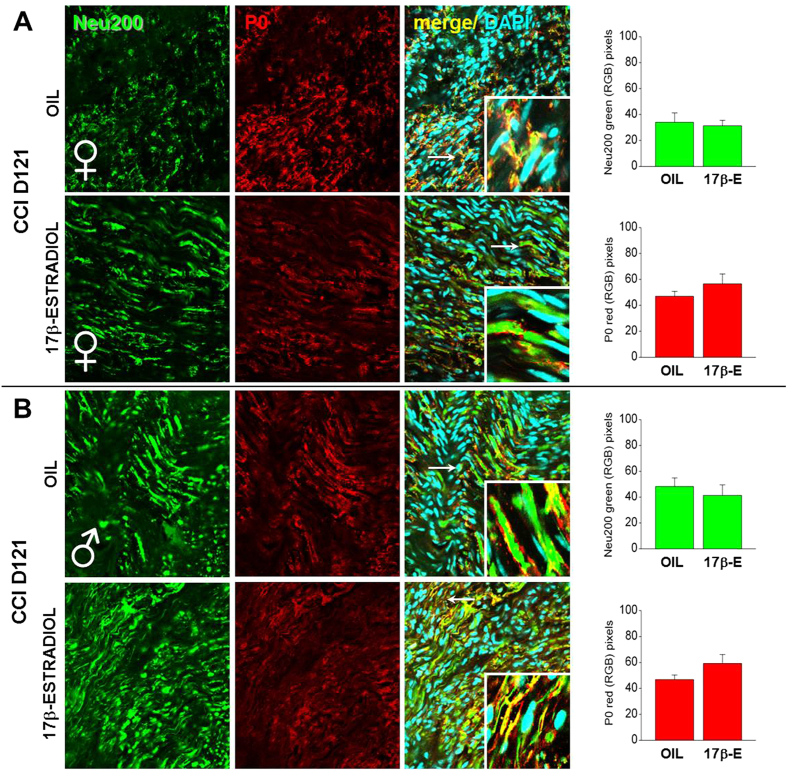
Sex-dependent differences in the expression and quantification of Neu200 and P0 in sciatic nerves at CCI D121. (**A,B**) Representative examples of high magnification (63X) IF images of Neu200 (green), P0 (red) and colocalization between Neu200 and P0 (merge; yellow), taken from sciatic nerves of OIL and 17β-estradiol female (**A**) and male (**B**) mice. Scale bar: 50 μm. White arrows indicate areas shown in the inset squares (63x, digital zoom 3). Right panels show quantification of Neu200 and P0 in OIL and 17β-estradiol mice by using RGB method that converted pixel in brightness values.

Immunofluorescence analysis was performed at CCI D7 and CCI D121. Figures 3 and 4 show IF analysis made in sections derived from sciatic nerve stained with antibodies against Neu200, the intermediate filaments marker that constitutes a cytoskeletal element of axons, permits to visualize regenerating fibres and plays an important physiological role for the formation and maintenance of the multilamellar structure of the myelin in the PNS, and P0, the major structural protein of peripheral myelin that determines the thickness of myelin^28^,^29^. By means of their co-staining and higher magnification the characterization and distribution of myelin and neurofilaments was examined.

Figure 3A shows higher expression of P0 in 17β-estradiol-treated females in comparison with OIL-treated female mice at CCI D7; the expression of Neu200 did not significantly change. The colocalization of these markers pointed out the differences in morphology and distribution between the two groups. In 17β-estradiol female mice P0 was detected in degenerating myelin sheaths and in myelin ovoids within the “digestion chamber of Cajal”, while, in OIL females P0 was minimally expressed, irregularly distributed and not completely formed in ovoids, suggesting a packing defect. Demyelination was evident in OIL female injured nerves since most of the myelin rings appeared collapsed and fragmented, in agreement with data previously reported^22^. Axon degeneration was also shown by the neurofilaments staining (Neu200-positive), which was not equally distributed along fibers. In OIL females, neurofilaments appeared thinner and the integrity of fibers was altered in comparison with 17β-estradiol females, which quite surprisingly show a partial nerve anatomical reconstitution/regeneration. Quantification of Neu200 and P0 expression is shown in histograms of Fig. 3A. Image analysis showed that, while there was an approximate 42% less immunoreactivity for P0 in OIL female nerves compared to 17β-estradiol females, indicating myelin loss, no significant differences were observed for Neu200.

Figure 3B shows Neu200 and P0 immunoreactivity in injured male mice. At CCI D7, the expression of Neu200 in OIL male group was significantly higher than in 17β-estradiol males, while SCs positive for the glycoprotein P0 were similarly expressed. The higher magnification shows that P0 was detected in “digestion chamber of Cajal” in both male groups, while Neu200 was better redistributed along fibres in 17β-estradiol males. Quantification by means of RGB method confirmed that in 17β-estradiol male mice there was a decrease of Neu200 expression in comparison with that observed in OIL males.

A higher expression of Neu200 and P0 were observed with WB analysis in 17β-estradiol females compared to OIL females, even if no significant differences were detected (Fig. 3C). As for males, WB confirmed the IF results showing a significant decrease in Neu200 expression in 17β-estradiol group (Fig. 3C).

We performed IF analysis also at the end of the behavioural test (CCI D121). Figure 4A shows similar expression of Neu200 and P0 in OIL- and 17β-estradiol treated female mice, but the higher magnification permitted to observe the different distribution of neurofilaments and myelin. In 17β-estradiol females the sciatic nerve appears highly regenerated in comparison with the sciatic nerve of OIL female mice, where fibres are highly disorganized, and neurofilaments short and segmented.

Differently from female groups, in males also high magnification did not show differences in the expression of Neu200 and P0 as well as in nerve reorganization (Fig. 4B). In fact, control male mice recovered at D81 and we observed an equal and similar distribution of neurofilaments and myelin along fibres.

#### Sex differences in the expression/activation of spinal neurons and glial cells

Behavioral results were also correlated with IF analyses of specific markers in the spinal cord. IF was performed at the same time-points as for sciatic nerves: CCI D7 and CCI D121. To analyze differences in the expression of neuronal and glial cells we performed IF staining of neurons (NeuN), astrocytes (GFAP) and microglia (CD11b), combined or not with phosphorylated p38 MAPKinase (p-p38) and with anti-Estrogen-related Receptor α (ERα).

Thanks to the colocalization with p-p38, immunohistological analysis permitted to put in evidence the activation of glial and neuronal cells in laminae I–IV of the lumbar spinal dorsal horns, favoring also a better appreciation of morphological features of neurons, astrocytes, and microglia and confirming differences across the experimental groups. On the other hand, the colocalization with ERα put in evidence the distribution of estrogen receptors on different cell types cells in the spinal cord.

We did not observe any difference in the expression of the neuronal marker at both low (10X) and high (63X) magnification in CCI females and males treated with 17β-estradiol or OIL. High magnification showed an increase in the colocalization of NeuN/ERα in CCI females and males treated with 17β-estradiol (see Supplementary Fig. S3).

**Figure 5:**
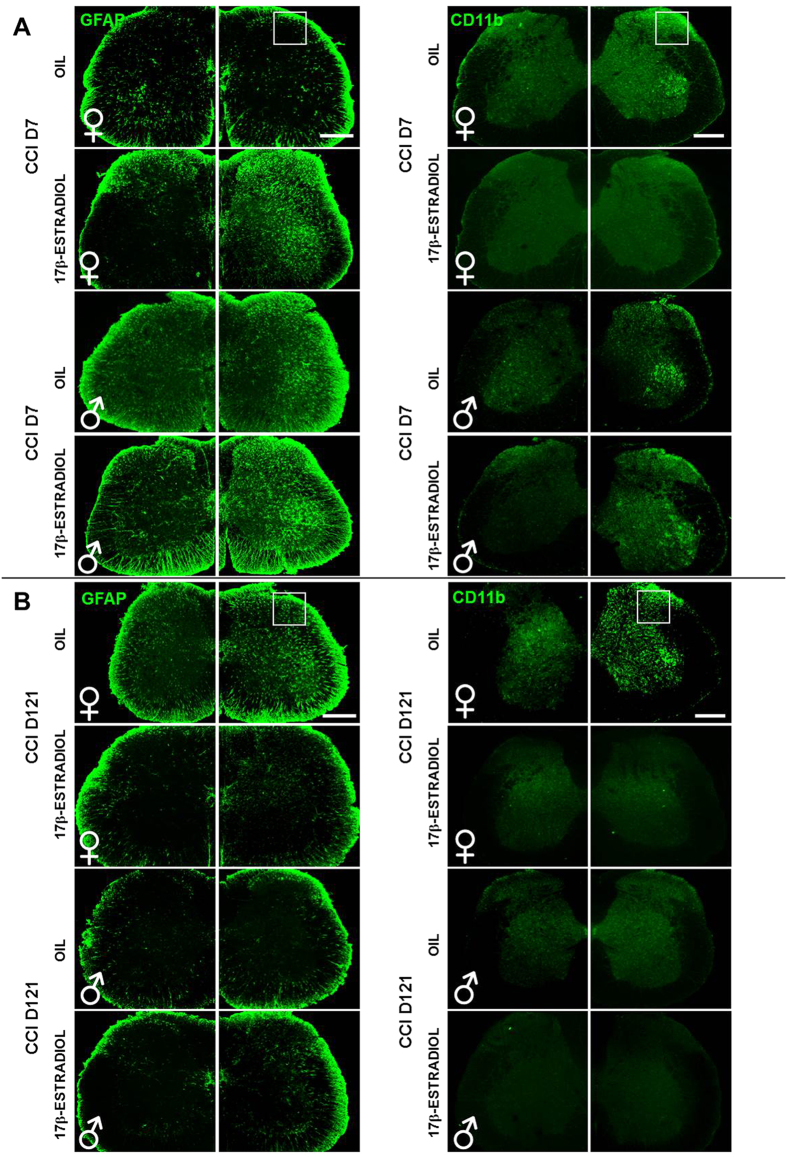
Sex-dependent differences in the expression/activation of spinal astrocytes and microglia, at CCI D7 and CCI D121. Representative examples of low magnification (10X) IF images of GFAP (astrocytes; green) and CD11b (microglia; green) in L4/L5 spinal cord sections taken from OIL and 17β-estradiol female and male mice at CCI D7 (**A**) and at CCI D121 (**B**). Scale bar: 300 μm. White squares indicate the ipsilateral dorsal horn considered for acquisition of the representative examples of high magnification (63X) IF images reported in Figs 6, 7, 8.

**Figure 6:**
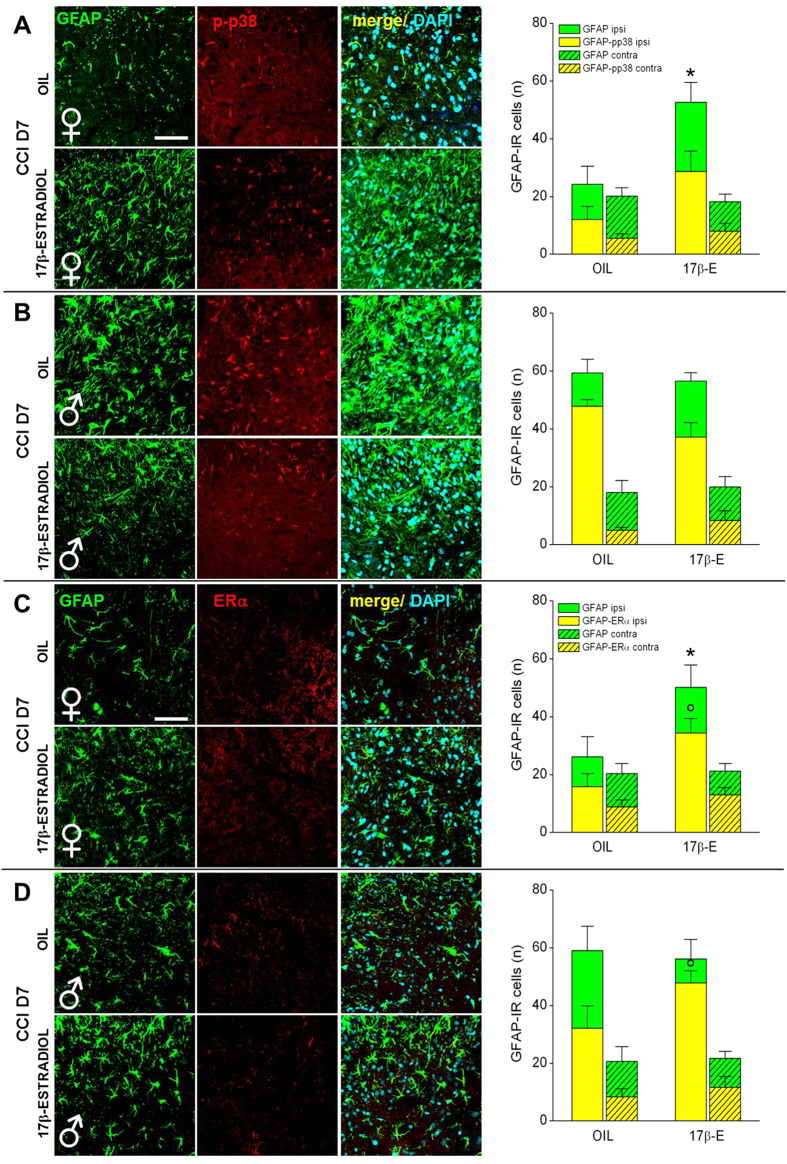
Sex-dependent differences in the expression/activation of spinal astrocytes at CCI D7. (**A,B**) Representative examples of high magnification (63X) IF images of GFAP (astrocytes; green), p-p38 (phosphorylated-p38, red), and their colocalization (merge; yellow) in L4/L5 spinal cord ipsilateral sections taken from OIL and 17β-estradiol female (**A**) and male (**B**) mice at CCI D7. Scale bar: 50 μm. Histograms show quantification of total number of GFAP-IR cells (green) and their colocalization with p-p38 (yellow) in OIL and 17β-estradiol (17β-E) female and male mice. Full columns indicate the side of spinal cord ipsilateral to the ligature, hatched columns indicate the contralateral side. (*)P < 0.05 vs OIL GFAP-IR. (**C,D**) Representative examples of high magnification (63X) IF images of GFAP (astrocytes; green), ERα (anti-Estrogen-related Receptor α; red), and their colocalization (merge: yellow) in L4/L5 spinal cord ipsilateral sections taken from OIL and 17β-estradiol female (C) and male (D) mice at CCI D7. Histograms show quantification of total number of GFAP-IR cells (green) and their colocalization with ERα (yellow) in OIL and 17β-estradiol (17β-E) female and male mice. Full columns indicate the side of spinal cord ipsilateral to the ligature, hatched columns indicate the contralateral side. (*)P < 0.05 vs OIL GFAP-IR; (°) P < 0.05 vs OIL GFAP-ERα-IR.

**Figure 7:**
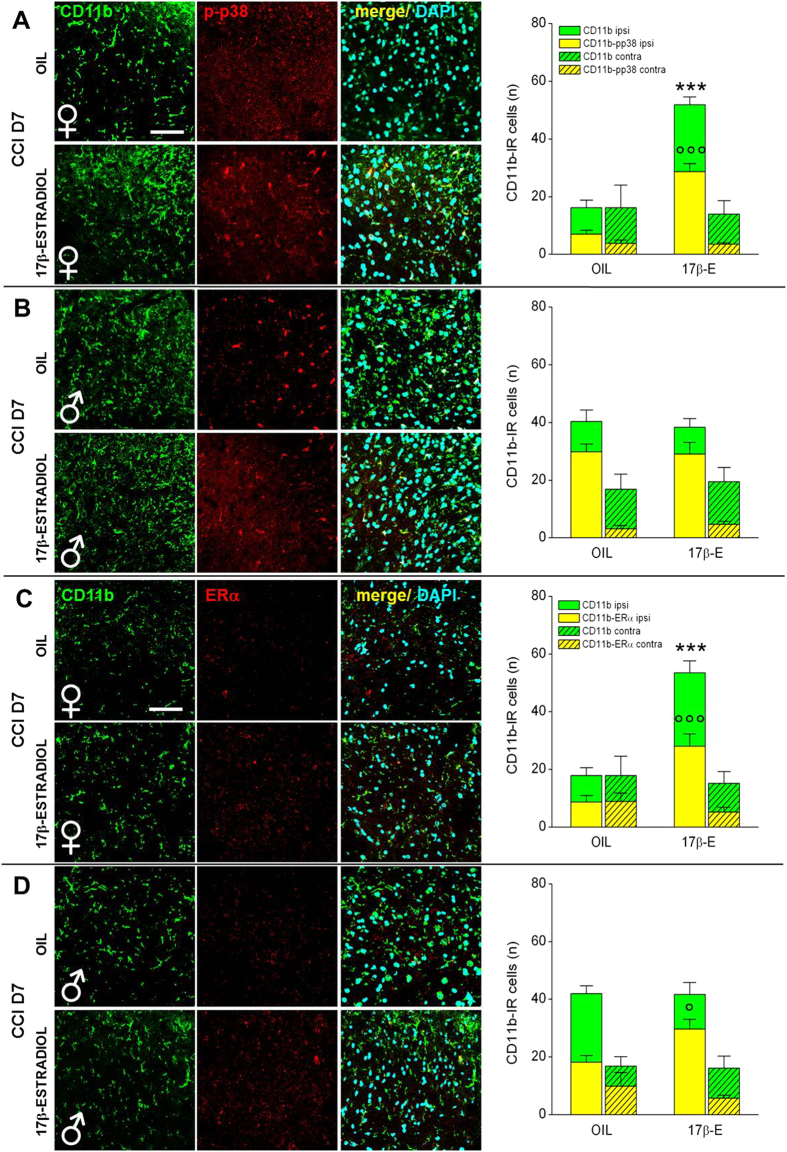
Sex-dependent differences in the expression/activation of spinal microglia at CCI D7. (**A,B**) Representative examples of high magnification (63X) IF images of CD11b (microglia; green), p-p38 (phosphorylated-p38; red), and their colocalization (merge, yellow) in L4/L5 spinal cord ipsilateral sections taken from OIL and 17β-estradiol female (**A**) and male (**B**) mice at CCI D7. Scale bar: 50 μm. Histograms show quantification of total number of CD11b-IR cells (green) and their colocalization with pp38 (yellow) in OIL and 17β-estradiol (17β-E) female and male mice. Full columns indicate the side of spinal cord ipsilateral to the ligature, hatched columns indicate the contralateral side. (***)P < 0.001 vs OIL CD11b-IR; (°°°) P < 0.001 vs OIL CD11b-pp38-IR. (**C,D**) Representative examples of high magnification (63X) IF images of CD11b (microglia; green), ERα (anti-Estrogen-related Receptor α; red), and their colocalization (merge; yellow) in L4/L5 spinal cord ipsilateral sections taken from OIL and 17β-estradiol female (**C**) and male (**D**) mice at CCI D7. Scale bar: 50 μm. Histograms show quantification of total number of CD11b-IR cells (green) and their colocalization with ERα (yellow) in OIL and 17β-estradiol (17β-E) female and male mice. Full columns indicate the side of spinal cord ipsilateral to the ligature, hatched columns indicate the contralateral side. (***)P < 0.001 vs OIL CD11b-IR; (°) P < 0.05, (°°°) P < 0.001 vs OIL CD11b- ERα-IR.

The results highlight important differences in the activation of glial cells between 17β-estradiol and OIL-treated female mice. Low and high magnification as well as quantification of the glial cells markers in spinal cord sections at CCI D7 show a significant increase of GFAP (Figs 5A and 6A,C) and CD11b expression (Figs 5A and 7A,C) in 17β-estradiol females compared to OIL females. Colocalization of glia with p-p38 and ERα resulted more pronounced in 17β-estradiol group. Altogether these data indicate that 17β-estradiol increases the proliferation of glial cells and regulates local inflammatory response.

**Figure 8:**
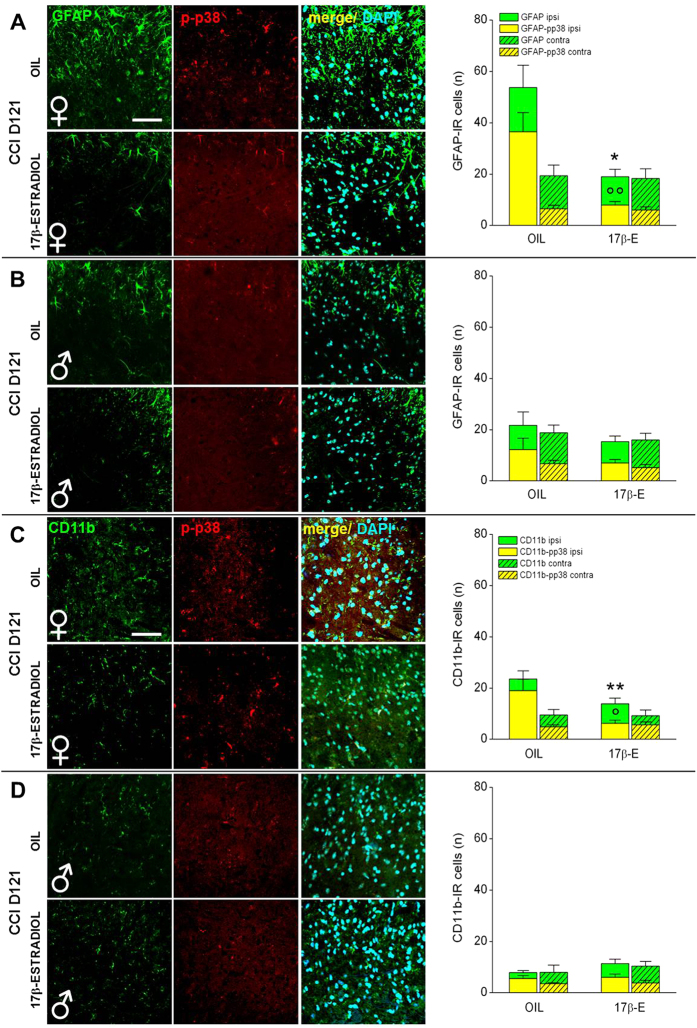
Sex-dependent differences in the expression/activation of spinal astrocytes and spinal microglia at CCI D121. (**A,B**) Representative examples of high magnification (63X) IF images of GFAP (astrocytes; green), p-p38 (phosphorylated-p38; red), and their colocalization (merge, yellow) in L4/L5 spinal cord ipsilateral sections taken from OIL and 17β-estradiol female (**A**) and male (**B**) mice at CCI D121. Scale bar: 50 μm. Histograms show the quantification of total number of GFAP IR cells (green) and their colocalization with pp38 (yellow) in OIL and 17β-estradiol (17β-E) female and male mice. Full columns indicate the side of spinal cord ipsilateral to the ligature, hatched columns indicate the contralateral side. (*)P < 0.05 vs OIL GFAP-IR; (°°) P < 0.01 vs OIL GFAP-pp38-IR. (**C,D**) Representative examples of high magnification (63X) IF images of CD11b (microglia; green), p-p38 (phosphorylated-p38; red), and their colocalization (merge, yellow) in L4/L5 spinal cord ipsilateral sections taken from OIL and 17β-estradiol female (**C**) and male (**D**) mice at CCI D121. Scale bar: 50 μm. Histograms show the quantification of total number of CD11b-IR cells (green) and their colocalization with pp38 (yellow) in OIL and 17β-estradiol (17β-E) female and male mice. Full columns indicate the side of spinal cord ipsilateral to the ligature, hatched columns indicate the contralateral side. (**)P < 0.01 vs OIL CD11b-IR; (°) P<0.05 vs OIL CD11b-pp-38-IR.

Histological analysis of spinal cord at CCI D121 (Figs 5B and 8A–C) confirmed several differences across the experimental female groups. Astrocytes and microglia were significantly more expressed in OIL females, which react with a long-lasting gliosis^30^, while in 17β-estradiol females the activation of astrocytes and microglia lasted few days. High magnification IF images, as well as quantification, show greater colocalization of GFAP and CD11b with p-p38 in OIL female than in 17β-estradiol female groups (Fig. 8A,C).

In 17β-estradiol-treated male mice, seven days after injury, we did not observe difference in the number and morphology of glial cells but attenuation of reactive gliosis in comparison with OIL-treated males (Figs 6B and 7B), which may justify the early complete recovery that occurred in 17β-estradiol males in comparison with OIL males. In 17β-estradiol-treated male mice we observed a higher colocalization of GFAP and CD11b with ERα in comparison with OIL-treated males (Figs 6D and 7D).

The staining performed at CCI D121 (Fig. 8B,D) revealed attenuation in reactive gliosis and absence of differences in the number of positive cells in male groups.

### Differential Proteomics and Bioinformatic Analysis

To further investigate the effects of 17β-estradiol treatment on sex-dependent differences in peripheral nerve regeneration, a comparative analysis of the protein repertoire was performed at CCI D7 and CCI D121. A label free differential proteomics experiment by data independent analysis was designed based on a shotgun discovery proteomics methodology^22^,^31^,^32^. The comparative analysis was performed, in both sexes, between 17β-estradiol- or OIL- treated mice. The OIL group was considered as the control reference group.

For females the proteomic analysis showed a treatment effect at CCI D7. A comparison of 17β-estradiol and OIL proteome of sciatic nerve in CCI D7 mice revealed 20 proteins differentially expressed: 12 up-regulated in 17β-estradiol females and 8 up-regulated in OIL females (Table 1). In particular, in 17β-estradiol female mice the following proteins, implicated in a number of function including inflammatory and regenerative processes, were more expressed: i) fibrinogen gamma (FGG), protein associated with inflammation, SCs activation, migration, and macrophage infiltration; ii) peroxiredoxin 5 (Prdx5), a member of peroxidases family participating in the regulation of redox signaling pathways; iii) keratin type II cytoskeletal 7 (Krt7), the cytoskeletal protein alpha internexin (Ina, a member of the class IV intermediate filament proteins) and myosin isoforms (Myh3, Myh7B, Myh8), proteins involved in morphogenesis and axonal transport; iv) myelin protein 0 (P0 -Mpz, a member of the immunoglobulin gene superfamily), the major protein component of peripheral nerve myelin. The higher expression of P0 in 17β-estradiol females confirms the results obtained by means of IF experiment. Another protein up-regulated in 17β-estradiol groups was apolipoprotein AI (Apoa I), whose up-regulation in 17β-estradiol females after nerve injury remarks its importance as transport vehicle for lipids between cells during neuronal degeneration and regeneration.

As far as OIL females are concerned, proteomic analysis revealed the following proteins up-regulated in comparison with 17β-estradiol mice: i) myosin isoforms (Myh1, Myh7); ii) annexin 5 (Anxa5), member of calcium-dependent phospholipid-binding protein family generally involved in immune and inflammatory reactions; iii) parvalbumin (Pvalb), a calcium-binding albumin protein; iv) 14-3-3 proteins zeta (Ywhaz), important mediators of anti-apoptotic signals, present in several types of neuron. In CCI OIL females we also observed an increased accumulation of proteins associated with metabolism, such as glucose 6-phosphate isomerase (Gpi).

Proteomic analysis of sciatic nerves taken from injured female mice at CCI D121 revealed 11 proteins differentially expressed in the two groups (Table 2) with the majority of the proteins expressed more in 17β-estradiol (7 in 17β-estradiol versus 4 in OIL). In 17β-estradiol females a higher expression of proteins involved in reorganization of SC, such as the myosin isoform Myh7 and Myh7B, tropomyosin alpha 1 and beta (Tpm1, Tpm2) and triosephosphate isomerase (Tpi), was observed. In OIL groups, there was a higher expression of alpha actinin 3, together with two myosin proteins (Myh6, Myh8) and serotransferrin (Tf), an iron carrier protein that plays a role in the maturation of SCs in physiological conditions.

We did not observe any relevant difference in the expression of proteins implicated in the processes object of our study between OIL- and 17β-estradiol-treated male mice, neither at CCI D7 nor at CCI D121 (data not shown).

## Discussion

In a previous study we compared female and male mice subjected to CCI and we observed a clear-cut sex-related difference in development and recovery from neuropathic pain: male mice showed a gradual decrease of CCI-induced allodynia reaching a complete recovery while in female mice allodynia was still present 121 days after surgery, at the end of the experiments^22^. In the present study we further investigated this sex-related difference on neuropathic pain and we demonstrated that the modulation of the ERs with their agonist 17β-estradiol lets to reduce this discrepancy making possible a recovery also for female mice.

Estrogen receptors are expressed by sensory neurons and in the dorsal horn of the spinal cord in the vicinity of nociceptive nerve terminals providing the means for a direct modulation of noxious input by gonadal hormones^33^. The classical estrogen action occurs through the entry of estrogen into the cell, interaction with the nuclear ERα and ERβ, and transcriptional activation of estrogen-responsive genes^34^. This cell signaling mechanism can take several hours or more to achieve its final downstream effects. In addition to classical genomic action, non-genomic estrogen-induced rapid cell signaling pathways via extranuclear ERs or estrogen G-protein coupled receptor GPR30 were also reported^18^ and recognized as important contributors to the overall biological response^17^.

The existing literature on the effects of estrogens is inconsistent; it is difficult to definitively define estrogens as “pro-nociceptive” or “anti-nociceptive” and it is possible that both effects may occur at different times depending on estrogen level and structures and systems involved^4^,^8^,^35^,^36^. The absence of estrogens has often been shown to result in increased pain sensitivity^16^; however, when estrogen levels are constantly elevated as in pregnancy, pain sensitivity is known to decrease^37^ and ovariectomy results in hyperalgesia in mice subjected to both mechanical and thermal tests^38^.

Differing from most experimental studies carried out in ovariectomized female rats and/or mice, we decided to investigate the modulatory effects of 17β-estradiol administration in intact animals affected by neuropathy. As a matter of fact, we were not especially interested in the compensatory mechanisms induced by a dysfunctional hormonal condition. Gonadectomy induces several morphological and functional changes in the CNS together with a hyperalgesic state of slow onset and long duration that can be reversed by estrogens^38^,^39^. Moreover, gonadectomy results in loss of behavioral and neuronal ‘adaptation’ observed in intact animals under pain conditions, e.g. the formalin-induced behavioural and neuronal responses^40^. It has also to be considered that we did not observe any influence of estrous cycle on responses to neuropathy induction. On the basis of these observations and our previous results showing a disability of reacquiring the hindlimb functionality in neuropathic female mice^22^, we were interested in discovering if exogenous administration of estrogens in neuropathic animals could be able to recover the pathological condition. The results obtained support our hypothesis.

According to our previous experiments, in the present study we observed that CCI-induced allodynia developed differently in males and females. More meaningfully, we demonstrated that 17β-estradiol, whose first injection started one hour after injury, was able to induce relief of pain in both female and male neuropathic animals after 7 days of repeated administrations. Mechanical allodynia, still present at the end of testing in control neuropathic female mice, was significantly attenuated in 17β-estradiol-treated females, reduction maintained during the overall time-course. In females, treatment with 17β-estradiol resulted in a complete recovery on D71 from CCI, while allodynia was still present at CCI D121 in vehicle-treated mice. Analgesia together with a facilitated recovery was observed also in male mice following 17β-estradiol administration.

Similar positive effects were obtained analyzing another behavioral response, weight bearing. Neuropathic females and males treated with the hormone show a faster recovery in comparison with vehicle-treated mice, which more slowly regained the equal loading of the hindlimbs.

Another important point emerging from our data highlights the important role of 17β-estradiol in neural regenerative processes. We observed relevant changes in the expression of important proteins associated with nerve injury and repair, such as P0 and Neu200, which play an important role for the formation and structure of myelin in the PNS. In 17β-estradiol females the sciatic nerve appears highly regenerated in comparison with the sciatic nerve of OIL female mice, where fibres are highly disorganized, and neurofilaments short and segmented.

Sciatic nerve regeneration involves several overlapping stages, including apoptosis and inflammation, Schwann cell activation/proliferation, and myelin remodeling. Faster process of regenerating nociceptive axons in 17β-estradiol treated animals than in other groups could be due to a greater capacity to improve the growth of nerve cell bodies and/or the growth promoting conditions in the degenerating nerve segment. At CCI D7, we found, in both sexes, an increase in ERα expression and in SC proliferation. SC are important not only in peripheral axons regeneration, but also in reinnervation. These data are in agreement with papers showing that ERα activation improves the ability of SC to proliferate and to guide the outgrowing axons, important for a correct regeneration^41^. Moreover, 17β-estradiol stimulates the degradation of P0 in degenerating myelin sheaths and in myelin ovoids within the “digestion chamber of Cajal”, crucial step for a correct degradation and recycling of myelin. The significant effect of 17β-estradiol in axon regeneration was further confirmed analyzing fiber morphology by means of immunofluorescence-staining: in both sexes we observed a better fiber integrity and regeneration, while in the control groups it appeared highly disorganized, shorter and segmented.

The effects of 17β-estradiol on myelination processes and axon outgrowth in neuropathic females were also supported by the proteomic analysis. In fact, we previously discovered that many myelin proteins having a role in the regenerative phase, such as the myelin protein P0, are significantly more expressed in male than in female neuropathic mice^22^. After estrogen treatment, at CCI D7, also females showed a significant enhancement in the expression of P0, as well as of some cytoskeletal proteins, when compared with OIL-treated mice. Interestingly, a previous proteomics investigation proposed a therapeutic use of β-estradiol in neurodegenerative diseases as a mean to induce autophagy^42^, being autophagy modulation suggested as a powerful approach to prevent neuropathic pain chronification^43^.

Another interesting result that needs to be investigated in further experiments concerns the higher expression in neuropathic mice of a protein associated with metabolism such as Glucose 6 phosphate isomerase, which has been reported to play a relevant role in rheumatoid arthritis^44^ and diabetic neuropathy^30^.

Inflammatory and associated immune responses are crucial for the development of neuropathic pain^45^,^46^, moreover a multiplicity of neuronal and non-neuronal downstream targets are affected by circulating estrogens^41^,^47^. Together with peripheral events, we observed central modulation of nerve injury-related phenomena. Histological analysis of glia markers in spinal cord sections revealed several differences across the experimental groups.

We have previously demonstrated that, differing from male mice where a reactive gliosis was present at CCI D7 but not at CCI D121, in females a later and chronic activation of glial cells occurred, still detectable 121 days after neuropathy induction^22^. The exaggerated and long-lasting activation of microglia and astrocytes may lead to neurotoxicity and may be detrimental for neural tissue regeneration^48^,^49^. Those results are confirmed also in this study; in fact, in OIL female group we observed the absence of glial activation at CCI D7, and the presence of long-lasting activation at the end of behavioral testing. Hormonal treatment modified the profile: 17β-estradiol-treated females show activation, morphological modification and proliferation of glial cells at CCI D7. These data may justify the early complete functional recovery that occurred in 17β-estradiol treated animals compared to control groups. In male mice, seven days after injury, we did not observe differences either in the number and morphology of glial cells and in reactive gliosis in 17β-estradiol males compared to OIL males.

Glial cells, astrocytes and microglia, are activated under the development of a neuropathic pain-like state in mice^50^. This condition is a graded phenomenon, characterized by specific morphological changes (hypertrophy), proliferation and changes in functional activities (migration to areas of damage, phagocytosis and production/release of pro-inflammatory substances). On one hand, activated microglia remove dead cells or dangerous debris either by releasing toxic factors (like tumor necrosis factor TNFα and interleukin IL-1β) or by phagocytosis; on the other hand, these cells repair injured cells by releasing neurotrophic factors, such as brain-derived neurotrophic factor (BDNF)^51^,^52^. Activated spinal glial cells lead to hyperalgesia and allodynia by releasing factors that act on neurons of the pain pathways. The present research confirms that steroid hormones, in particular 17β-estradiol, control reactive gliosis modifying the number of reactive astrocytes and reactive microglia and the expression of proinflammatory mediators^53^,^54^.

In conclusion, our results demonstrate the complex interactions involved in pain modulation induced by estrogens and reveal the powerful effect of 17β-estradiol in affecting neuropathic pain behavior, effect that becomes critical in female mice, which show a lack of recovery after the induction of neuropathy.

## Methods

### Animals

CD1 male and female mice, about 3 months old from Charles River Labs (Como, Italy) were used. Animals were housed in standard transparent plastic cages, in groups of 4, lined with sawdust under a standard 12/12-h light/dark cycle (07:00AM/07:00PM), with food and water available *ad libitum*. Testing was performed blind as for treatment group to which each subject belonged. After behavioral testing, the oestrous cycle was analyzed in females by means of vaginal smears. Because we did not observe any difference in the behavioral responses, we included all females in the same experimental group independently from the oestrous cycle. The experiments were approved by the Italian Ministry of Health with the authorization n. 187/2011 issued on September 28^th^ 2011, valid until September 28^th^ 2014, in accordance with the guidelines and regulations of the Italian Law (DLGs n.116, 27/1/1992), and carried out in that time interval. All procedures were in strict accordance with the current European and Italian National law (DLGs n.26, 04/03/2014, application of the European Communities Council Directive 2010/63/UE) on the use of animal for research and with the guidelines of the Committee for Research and Ethical Issues of IASP^55^.

### Surgery

Following the procedure originally proposed by Bennett and Xie^56^ adapted to the mouse, the Chronic Constriction Injury (CCI) model was used as model of neuropathic pain. CCI of sciatic nerve was performed under anaesthesia with chloral hydrate (500 mg/kg intraperitoneally; Sigma-Aldrich, Milan, Italy); the middle third of the right sciatic nerve was exposed through a 1.5 cm longitudinal skin incision. Three ligatures (5-0 chromic gut, Ethicon, Rome, Italy) were tied loosely around the sciatic nerve. The wound was then closed with 4-0 silk suture. In the following, the injured right hindpaw will be named as ipsilateral paw and the uninjured left hindpaw will be named as contralateral paw.

### Experimental groups

One hour after CCI male and female mice were randomly assigned to one of the following treatments (10 animals per group): i) 17β-estradiol group: treated with 17β-estradiol (Sigma-Aldrich), 50 μg/kg/day^57^; ii) OIL group (vehicle): treated with sweet almond oil, in an equivalent volume as per the preceding groups. Drugs were subcutaneously (s.c.) administered. The injections were performed daily, for seven days, from D0 to D6 after CCI.

### Estradiol plasma levels

Twenty-four hours after the last s.c. injection of 17β-estradiol two blood samples for each experimental groups were collected for measuring the levels of estradiol in serum and for comparing with the levels in naïve animals. Blood was collected from the abdominal aorta anaesthetized animals at the end of the experiment. It was centrifuged (3000 g for 15 min at 4 °C) to obtain serum. Samples were then frozen at –20 °C until the assay. Estradiol serum levels were determined by Charles River Research Animal Diagnostic Server (CR RADS) with Abcam Estradiol ELISA Assay Kit.

### Behavioral testing

#### Mechanical nociceptive threshold (Dynamic plantar aesthesiometer test)

The onset of neuropathy was assessed by measuring the sensitivity of both ipsilateral and contralateral hindpaws to normally non-noxious punctuate mechanical stimuli, at different time intervals from D3 to D121. The nerve injury-induced mechanical allodynia was tested by using a Dynamic Plantar Aesthesiometer (Ugo Basile, Model 37400), an apparatus that generates a mechanical force linearly increasing with time. The force is applied to the plantar surface of the mice hindpaw, and the nociceptive threshold is defined as the force, in grams, at which the mouse withdraws its paw. At each day of testing, the mechanical withdrawal response of ipsilateral and contralateral hindpaws was recorded for 3 consecutive trials with at least 10 seconds between each trial. The withdrawal threshold was taken to be the mean of the 3 trials. Preliminary experiments were also carried out to assess the effects of 17β-estradiol on locomotor behavior, measured as number of crossing from one compartment to the other in a toggle-floor box^58^. The locomotor activity was recorded for one hour in the experimental groups at D7 post CCI.

#### Weight bearing (incapacitance test)

Hind limb weight bearing was determined by using an Incapacitance apparatus (Linton Instrumentation, Norfolk, UK) consisting of a dual weight average. The apparatus is adapted for mice with a strain gauge/amplifier resolution of 0.03 g and strain gauge/amplifier accuracy of 0.1 g. Weight distribution was measured between ipsi- and contralateral hind limbs as previously described^22^. The weight percent distribution onto the ipsilateral hind limb was calculated by the following equation: [ipsilateral weight / (ipsilateral weight + contralateral weight)] × 100.

### Immunohistochemical analysis

Sciatic nerve and lumbar spinal cord (L4-L5) of mice belonging to each experimental group (n = 3/group) were harvested for IF analysis. Animals were sacrificed with lethal dose of chloral hydrate and perfused with saline followed by 4% paraformaldehyde in phosphate buffer saline (PBS, pH 7.4). Sciatic nerve and spinal cord were removed and kept in immersion for 48 h in 4% paraformaldehyde in phosphate buffer saline (PBS, pH 7.4), after cryoprotection with solution of 30% (w/v) sucrose in PBS and maintained at −80 °C. Cryostat sections of sciatic nerve and spinal cord, 20 and 40 μm respectively, were taken.

For sciatic nerve, IF analysis was made in CCI mice at two different time points, at CCI D7 and CCI D121, in males and females treated with OIL or 17β-estradiol. For double IF staining, sections were incubated overnight with: i) anti-S100β (mouse monoclonal, 1:100, S2532; Sigma-Aldrich), and anti-Estrogen-related Receptor α (ERα, rabbit polyclonal, 1:100, 17-603; Millipore, Billerica, MA, USA) antibodies; or ii) anti-Neurofilament 200 (Neu200, rabbit polyclonal, 1:100, N4142; Sigma-Aldrich) and anti-myelin protein zero (P0 or MPZ, chicken polyclonal, 1:100, AB9352; Millipore) antibodies. Both antibodies were diluted in Triton 0,3% (Sigma-Aldrich).

For spinal cord, IF analysis was made in CCI D7 and CCI D121 mice. For double IF staining, sections of lumbar spinal cord were incubated for 48 h at room temperature with: anti-GFAP (glial fibrillary acidic protein, astrocytes marker) antibody (mouse monoclonal, 1:100, Sigma-Aldrich: G6171); anti-CD11b (complement receptor 3/cluster of differentiation 11b, microglia marker) antibody (rat anti-mouse, 1:100, Millipore: MCA711), anti-NeuN (neuronal marker) antibody (mouse monoclonal, 1:100, MAB377; AbD Serotec, Kidlington, UK); anti-p-p38 antibody (rabbit polyclonal, 1:100, sc-28533; Santa Cruz Biotechnology) and anti-Estrogen-related Receptor α (ERα, rabbit polyclonal, 1:100, 17-603; Millipore, Billerica, MA, USA) antibodies in Triton 0,3%.

After three washings in PBS, sciatic nerve or spinal cord sections were incubated for 2 h at room temperature with fluorescein-conjugated donkey anti-mouse (ALEXA Fluor 488, 1:100, Jackson ImmunoResearch), fluorescein conjugated rat anti-mouse (FITC, 1:100, Jackson ImmunoResearch) or rhodamine conjugated goat anti-rabbit (TRITC, 1:100, Jackson ImmunoResearch) secondary antibodies in 0.3% Triton. After two washings in PBS, sections were incubated for 10 minutes with bisBenzimide, DNA-fluorochrome (Hoechst, 1:1000, Sigma-Aldrich) in PBS.

### Cell quantification

Images of immunostained sections were acquired by laser scanning confocal microscopy using a TCS SP5 microscope (Leica Microsystem) connected to digital camera diagnostic instruments operated by I.A.S. software of Delta Systems Italia. Figures were assembled by using Adobe Photoshop CS3 and Adobe Illustrator 10. Quantification was performed by using the ImageJ software (version 1.41, National Institutes of Health, USA). The number of positive cells was automatically counted (two sections/animal) with a mark and count tool and for each group of mice the mean of three animals was calculated.

High (63X) magnification images of immunostained sections of sciatic nerves were acquired and the number of Hoechst-positive cells was automatically counted. The fluorescence of all different markers was quantified with RGB (Red, Green and Blue) method that is largely applied to detect, digitalize and analyze microscopic images from biological samples by confocal microscope^59^. The number of pixels of each color was automatically counted and then converted to a brightness value, using ImageJ software (http://rsb.info.nih.gov/ij/).

Low (10X) and high (63X) magnification images of immunostained sections of spinal cord were acquired. To quantify the immunoreactivity of astrocytes, microglia and neurons in the spinal cord, high magnification images of the ipsilateral side of the dorsal horns for each animal were captured with a 63X objective at zoom factor 1 by using a constant set of acquisition parameters. Identification of laminae area was facilitated by means of Hoechst staining of cell nuclei. For analysis of labelling, the entire image (a square of 230 μm of side) of the ipsilateral side of spinal cord was quantified by counting number of positive immunoreactive (IR) cells. A similar procedure was adopted for quantification of IR cells and colocalization with p-p38 and ERα in high magnification images.

### Protein Purification and label free differential proteomic shotgun analysis

The analysis was performed on total proteins extracted from sciatic nerves obtained from different groups of animals (n = 3 mice/group): male and female, OIL and 17β-estradiol. The tissues are collected from CCI D7 and CCI D121 mice. Nerves obtained from mice subjected to the same treatment were pooled together. Each pool was immersed in a denaturing buffer (6M Urea in 100 mM Tris-Cl pH 7.8) and manually fractionated with a mini-potter. The samples were then sonicated in a water bath (Ultrasonic Cleaner, CP104, EIA) at 95% US power, at room temperature for 15 minutes, passed through a syringe with a 22GA 11/4 IN 0.7 × 30 needle and centrifuged at 13000 rpm at 4 °C for 30 min. Protein concentrations were determined in each supernatant and 25 μg of total protein per pool was subjected to trypsin digestion as previously described^31^,^32^.

Upon digestion, 0.260 μg/μL of triptic peptides solution containing 100 fmol/μL of Yeast Enolase digestion (SwissProt P00924) (Waters, Milford, MA, USA,) added as internal standard was used for label free differential proteomic shotgun analysis by means of Ultra Performance Liquid Chromatography on a nanoACQUITY UPLC System (Waters), coupled to a hybrid quadruple orthogonal acceleration Time-of-flight Mass Spectrometer (Q-Tof Premier, Waters Corp). Mass spectrometry data were acquired in MS^E^ (Expression mode: data independent parallel parent and fragment ion analysis). Continuum LC-MS data from three replicate experiments for each pool were processed for qualitative and quantitative analysis using the software ProteinLynx Global Server v. 2.4 (PLGS, Waters). The qualitative identification of proteins was obtained by searching in a mouse database (UniProt KB/Swiss-Prot Protein Knowledgebase release 2012_11 of 28-November-12 containing 538585 sequence entries, taxonomical restrictions: Mus Musculus, 16573 sequence entries) to which data from *Saccharomyces cerevisiae* Enolase (accession number: P00924) were appended. Identified proteins were normalized against P00924 entry in the differential quantitative analysis (expression analysis). Our data have been qualified to detect a difference in relative ratio larger than 20%; however, to keep a conservative approach, we filtered our protein hits to a fold difference larger than 30% (corresponding to a ratio of + or −1.3). These performances are in line with those reported in previous papers from our group and others^31^,^32^.

### Protein isolation and Western blot analysis

Sciatic nerves from OIL- and 17β-estradiol-treated male and female mice (n = 3 mice/group) were taken at D7. Nerves deriving from the same experimental group were pooled together and homogenized in lysis buffer (320 mM sucrose, 10% glycerol, 50 mM NaCl, 50 mM Tris-HCl pH7.5, 1% Triton X-100, plus protease inhibitor cocktail Sigma-P8340), sonicated in a water bath (Ultrasonic Cleaner, CP104, EIA) at 95% US power, 5 min. at RT, incubated on ice for 30 min. and centrifuged at 13,000 rpm for 20 minutes to remove tissue debris. The total protein content of resulting supernatant was determined. Proteins were applied to sodium dodecyl sulfate–polyacrylamide gel electrophoresis and electroblotted on a nitrocellulose membrane. Immunoblotting analysis was performed using a chemiluminescence detection kit.

The relative levels of immunoreactivity were determined by densitometry using the ImageJ software (version 1.41, National Institutes of Health, USA). Samples were incubated with the following primary antibodies: rabbit polyclonal anti-Neurofilament 200 (Neu200, rabbit polyclonal, 1:100, N4142; Sigma-Aldrich); anti-myelin protein zero (P0 or MPZ, chicken polyclonal, 1:100, AB9352; Millipore) and Anti-β-Actin Clone AC-15(mouse monoclonal, 1:5000, A5441; Sigma-Aldrich).

### Data analysis

All values are expressed as mean ± SEM. Two-way ANOVAs for repeated measures were used to analyze the effects of sex-related behavioral differences; when necessary, post hoc comparisons were carried out using Tukey-Kramer test. The statistical comparisons concerning immunohistochemical data were carried out by means of Student’s T-test for independent samples. Differences were considered significant at P < 0.05.

## Additional Information

**How to cite this article**: Vacca, V. *et al.* 17beta-estradiol counteracts neuropathic pain: a behavioural, immunohistochemical, and proteomic investigation on sex-related differences in mice. *Sci. Rep.* **6**, 18980; doi: 10.1038/srep18980 (2016).

## Supplementary Material

Supplementary Information

## Figures and Tables

**Table 1:** Proteins differentially expressed in OIL and 17β-estradiol female CCI D7 mice.

Accession^a^	Description (Gene Name)	Highly represented^b^	OIL:17β-E ratio^c^	Location^d^	Type(s)^d^
Q8VCM7	Fibrinogen gamma chain OS Mus musculus GN Fgg PE 2 SV 1	17β-estradiol*	0,1	Cytoplasm	other
P99029	Peroxiredoxin 5 mitochondrial OS Mus musculus GN Prdx5 PE 1 SV 2	17β-estradiol*	0,1	Plasma Membrane	other
Q9DCV7	Keratin type II cytoskeletal 7 OS Mus musculus GN Krt7 PE 1 SV 1	17β-estradiol*	0,1	Extracell Space	other
Q8CGP0	Histone H2B type 3 B OS Mus musculus GN Hist3h2bb PE 1 SV 3	17β-estradiol*	0,1	Extracell Space	other
P13541	Myosin 3 OS Mus musculus GN Myh3 PE 2 SV 2		0,23	Cytoplasm	transporter
P46660	Alpha internexin OS Mus musculus GN Ina PE 1 SV 2		0,31	Cytoplasm	other
P13542	Myosin 8 OS Mus musculus GN Myh8 PE 1 SV 2		0,38	Cytoplasm	other
P27573	Myelin protein P0 OS Mus musculus GN Mpz PE 1 SV 1		0,54	Cytoplasm	other
A2AQP0	Myosin 7B OS Mus musculus GN Myh7b PE 3 SV 1		0,57	Plasma Membrane	other
P07759	Serine protease inhibitor A3K OS Mus musculus GN Serpina3k PE 1 SV 2		0,64	Cytoplasm	enzyme
Q00623	Apolipoprotein A I OS Mus musculus GN Apoa1 PE 1 SV 2		0,67	Cytoplasm	other
P07724	Serum albumin OS Mus musculus GN Alb PE 1 SV 3		0,7	Extracell Space	other
Q5SX40	Myosin 1 OS Mus musculus GN Myh1 PE 1 SV 1		2,69	Cytoplasm	enzyme
Q91Z83	Myosin 7 OS Mus musculus GN Myh7 PE 1 SV 1		3,1	Extracell Space	transporter
P32848	Parvalbumin alpha OS Mus musculus GN Pvalb PE 1 SV 3	OIL^†^	10	Cytoplasm	enzyme
P70296	Phosphatidylethanolamine binding protein 1 OS Mus musculus GN Pebp1 PE 1 SV 3	OIL^†^	10	Extracell Space	other
P48036	Annexin A5 OS Mus musculus GN Anxa5 PE 1 SV 1	OIL^†^	10	Cytoplasm	other
P06745	Glucose 6 phosphate isomerase OS Mus musculus GN Gpi PE 1 SV 4	OIL^†^	10	unknown	other
P63101	14 3 3 protein zeta delta OS Mus musculus GN Ywhaz PE 1 SV 1	OIL^†^	10	Extracell Space	other
O88990	Alpha actinin 3 OS Mus musculus GN Actn3 PE 2 SV 1	OIL^†^	10	Extracell Space	transporter

Proteins upregulated (†) and downregulated (*) in OIL vs 17β-estradiol female CCI D7 nerves.

^a^Unique protein sequence identifier according to UniProtKB/Swiss-Prot 2012-11, Rel. November 8, 2012, restricted to Mus Musculus Taxonomy.

^b^Protein found highly represented in the indicated group. Highly represented proteins are those that in the Differential Proteomics Analysis have been detected in only one group (treated with OIL or 17β-estradiol in this case) and appeared in every injection for that group. In this work we associated to them an arbitrary ratio value equal to 10 fold or 0.1 fold.

^c^Ratio of expression between 2 experimental groups.

^d^According to Ingenuity Pathways Analysis (Ingenuity® Systems, Build version 212183, Content version 1240262, Release date 2012/03/09, www.ingenuity.com).

**Table 2:** Proteins differentially expressed in OIL and 17β-estradiol female CCI D121 mice.

Accession^a^	Description (Gene Name)	Highly represented^b^	OIL:17β-E ratio^c^	Location^d^	Type(s)^d^
Q91Z83	Myosin 7 OS Mus musculus GN Myh7 PE 1 SV 1		0,16	Cytoplasm	other
P58774	Tropomyosin beta chain OS Mus musculus GN Tpm2 PE 1 SV 1		0,5	Plasma Membrane	other
A2AQP0	Myosin 7B OS Mus musculus GN Myh7b PE 3 SV 1		0,58	Extracell Space	other
P58771	Tropomyosin alpha 1 chain OS Mus musculus GN Tpm1 PE 1 SV 1		0,63	Extracell Space	other
P97457	Myosin regulatory light chain 2 skeletal muscle isoform OS Mus musculus GN Mylpf PE 1 SV 3		0,71	Cytoplasm	transporter
P05977	Myosin light chain 1 3 skeletal muscle isoform OS Mus musculus GN Myl1 PE 1 SV 2		0,71	Cytoplasm	other
P17751	Triosephosphate isomerase OS Mus musculus GN Tpi1 PE 1 SV 4		0,72	Cytoplasm	other
Q921I1	Serotransferrin OS Mus musculus GN Tf PE 1 SV 1		1,38	Cytoplasm	other
P13542	Myosin 8 OS Mus musculus GN Myh8 PE 1 SV 2		2,12	Plasma Membrane	other
Q02566	Myosin 6 OS Mus musculus GN Myh6 PE 1 SV 2		3,22	Cytoplasm	enzyme
O88990	Alpha actinin 3 OS Mus musculus GN Actn3 PE 2 SV 1	OIL^†^	10	Cytoplasm	other

Proteins upregulated (†) and downregulated (*) in OIL vs 17β-estradiol female CCI D121 nerves.

^a^Unique protein sequence identifier according to UniProtKB/Swiss-Prot 2012-11, Rel. November 28, 2012, restricted to Mus Musculus Taxonomy.

^b^Protein found highly represented in the indicated group. Highly represented proteins are those that in the Differential Proteomics Analysis have been detected in only one group (treated with OIL or 17β-estradiol in this case) and appeared in every injection for that group. In this work we associated to them an arbitrary ratio value equal to 10 fold or 0.1 fold.

^c^Ratio of expression between 2 experimental groups.

^d^According to Ingenuity Pathways Analysis (Ingenuity® Systems, Build version 212183, Content version 1240262, Release date 2012/03/09, www.ingenuity.com).
