# Supplementary material for: 17beta-estradiol counteracts neuropathic pain: a behavioural, immunohistochemical, and proteomic investigation on sex-related differences in mice
Source: Sci Rep. 2016 Jan 8;6:18980. doi: 10.1038/srep18980 (PMC4705539; doi:10.1038/srep18980)
Supplement: Supplementary Information [file srep18980-s1.pdf]

Supplementary Information for

17beta-estradiol counteracts neuropathic pain: a behavioural, immunohistochemical, and proteomic investigation on sex-related differences in mice.

Valentina Vacca [a,b], Sara Marinelli [a,b], Luisa Pieroni [b,c], Andrea Urbani [b,c], Siro Luvisetto [a,b] and Flaminia Pavone [a,b*]

[a] CNR - National Research Council, Institute of Cell Biology and Neurobiology, 00143 Roma, Italy.

[b] IRCCS Fondazione Santa Lucia, 00143 Roma, Italy.

[c] Department of Experimental Medicine and Surgery, Division of Biochemistry, University of "Tor Vergata", 00133 Roma, Italy.

[*] Corresponding author at: CNR – National Research Council, Institute of Cell Biology and Neurobiology, Via Fosso di Fiorano 64, Roma 00143, Italy.

Tel.: +39 06 50170 3271; fax: +39 06 50170 3304.

E-mail address: flaminia.pavone@cnr.it

Supplementary Figure 1

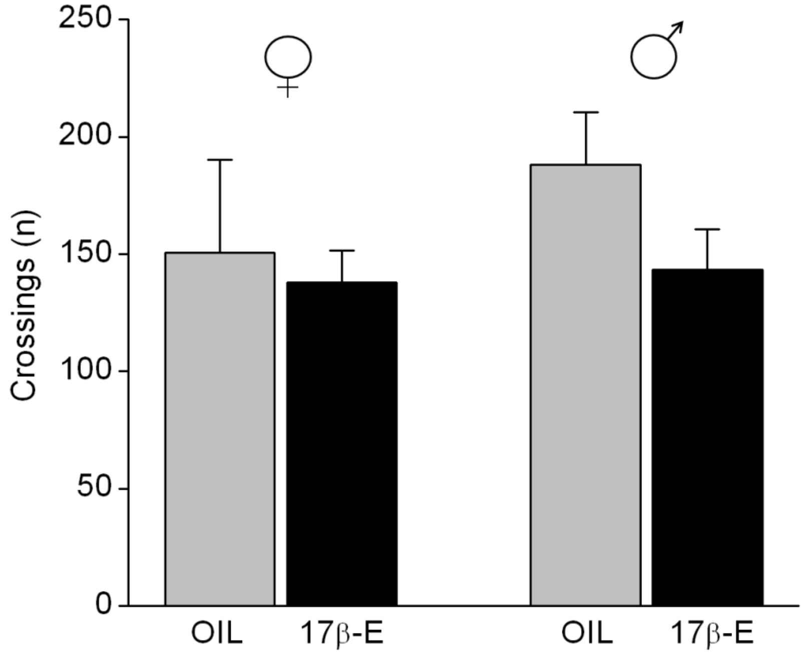

Supplementary Figure 1 | Effects of 17b-estradiol on locomotor activity.

Histograms show quantification of total number of crossing from one compartment to the other of a toggle-floor box, recorded for one hour. Left columns show locomotor activity of female in OIL (gray) and 17β-estradiol (17β-E; black) groups. Right columns show locomotor activity of male in OIL (gray) and 17β-estradiol (17β-E; black) groups. Each experimental group was composed of 6 mice.

Supplementary Figure 2

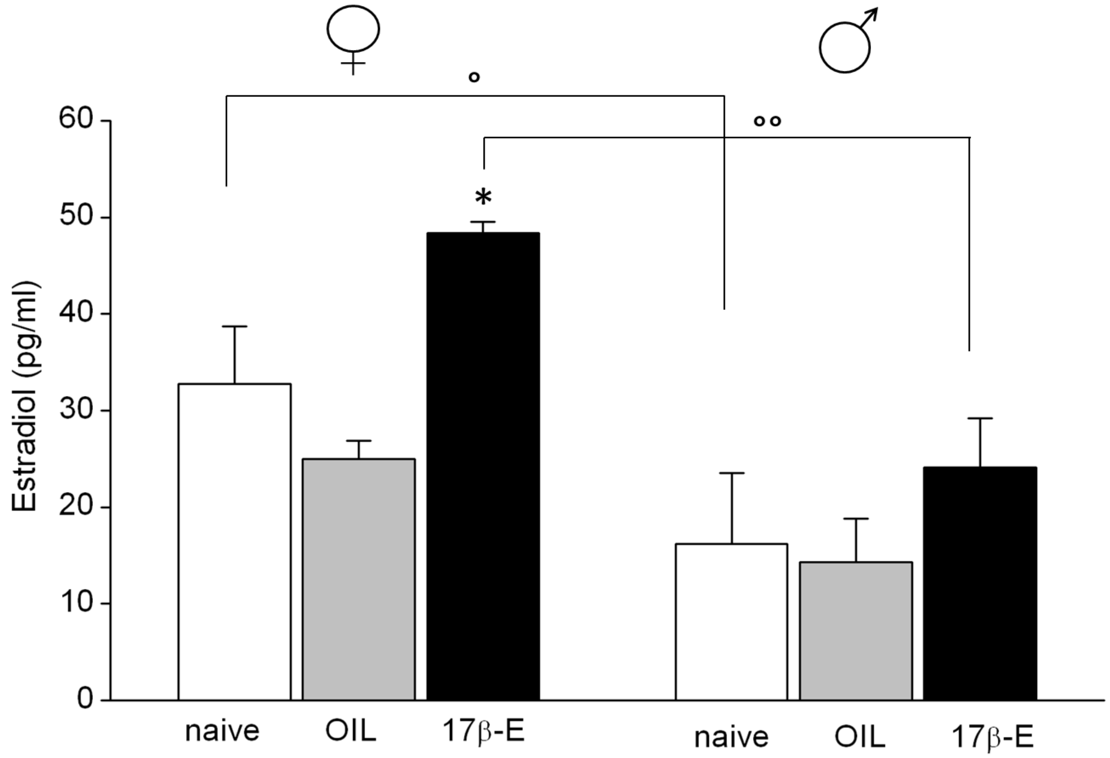

Supplementary Figure 2 | Levels of Estradiol in serum of naïve, OIL- and 17β-estradiol-treated mice.

Left columns show estradiol serum levels of naive (white) and of neuropathic female mice treated with OIL (gray) or 17β-estradiol (17β-E; black). Right columns show estradiol serum levels of naive (white) and of neuropathic male mice treated with OIL (gray) and 17β-estradiol (17β-E; black). Number of mice was 2 for each experimental group. ANOVA one-way showed a significant effect of treatment in females ($F_{2,3}$=10.598, P<0.05), while no significant effect was observed in male mice ($F_{2,3}$=0.814, P=0.521). (*) P<0.05 vs OIL; (°) P<0.05, (°°) P<0.01 female vs male mice.

Supplementary Figure 3

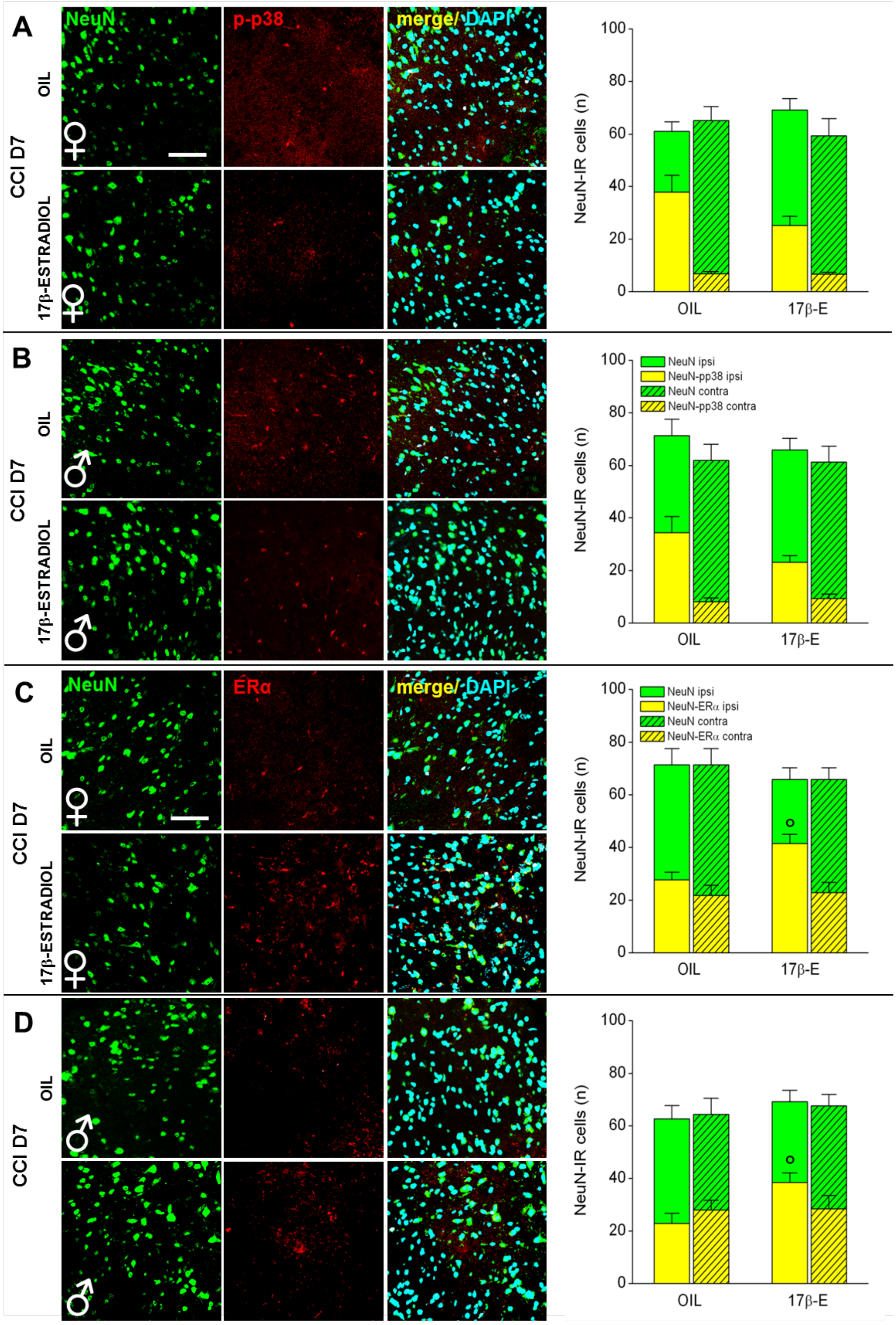

Supplementary Figure 3 | Sex-dependent differences in the expression/activation of

neurons in spinal cord at CCI D7.

Representative examples of high magnification (63X) IF images of NeuN (neurons; green), p-p38 (phosphorylated (p)-p38 ,red) and their colocalization (merge, yellow) (A and B) and ERα (anti-Estrogen-related Receptor α; red) and their colocalization (merge; yellow) (C and D) in L4/L5 ipsilateral spinal cord sections taken from OIL or 17β-estradiol female (A, C) and male (B, D) mice at CCI D7. Scale bar: 50 μm.

Histograms show quantification of total number of NeuN-IR cells (green) and their colocalization with p-p38 or ERα (yellow) in OIL and 17β-estradiol female and male mice. Full columns indicate ipsilateral side of spinal cord, while hatched columns indicate contralateral side of spinal cord. (°) $P<0.05$ vs OIL NeuN- ERα IR.
